# Effects of molecular characteristics and microstructure of amaranth particle sizes on dough rheology and wheat bread characteristics

**DOI:** 10.1038/s41598-022-12017-7

**Published:** 2022-05-12

**Authors:** Ionica Coţovanu, Silvia Mironeasa

**Affiliations:** Faculty of Food Engineering, Ștefan cel Mare University of Suceava, 13 Universitatii Street, 720229 Suceava, Romania

**Keywords:** Biotechnology, Plant biotechnology, Chemical engineering

## Abstract

The aim of this research is to investigate the molecular features and microstructure of amaranth flour (AF) fractions and their partial replacement effect of wheat flour (WF) on the chemical composition, dough dynamic rheology, technological and sensory characteristics of bread. The microstructure and molecular characteristics of AF were depending on their particle size (PS). When WF replacement increased and PS decreased the composite flour was richest in protein, lipids, and ash, while the moisture and carbohydrates of these flours decreased. Dynamic rheological behavior revealed significant variations depending on PS and replacement level. Bread volume and firmness increased when more than 15% AF from large PS replaced WF, while medium and small PS at 5–15% replacements was increased the loaf porosity. Chroma values decreased and samples became darker when the replacement level increased. Moreover, replacement of WF with various AF fractions impacted bread sensory characteristics, obtaining better acceptance for large and medium PS up to 10%.

## Introduction

In recent years, consumers have focused on their nutrition and health, giving increased attention to lifestyle. Thereby, interest in improveming the foods has been increased. Bakery products are considered the best products for improving the nutritional process due to the daily caloric requirement given by bread (more than 50% of energy intake)^1^. One of the solutions is a partial replacement of wheat flour with other raw materials, rich in nutritional ingredients. Composite flour technology represents the operations of mixing wheat with non-wheat flour which contains high protein components for use in bakery technology^2^. An important class of raw materials is pseudocereals like amaranth which has higher nutritional quality than that of cereals. Amaranth seeds contain relatively higher levels of protein (12.5‒20%) than common cereals (corn: 8.9–12.9%, wheat: 9.1–14.0%, oats: 16.0%, rice: 7.5–8.7%)^3^, some authors found that the proportion of amaranth protein is similar to that of soy^4^. According to Osborne's classification, amaranth proteins consist of about 40% albumin, 20% globulin, 25–30% gluteline, and only 2–3% prolamine^5^. It contains a high level of protein with a balanced amino-acids profile, lipids, vitamins, minerals, and bioactive compounds^6^. Amaranth seeds proteins are rich in lysine, threonine, and methionine and encrypted peptides with various biological functions which have health benefits for the consumers^7^. Lipids contain a high level of unsaturated fatty acids such as palmitic, oleic, linoleic, and linolenic acids and tocotrienols and squalene, with an important role in lowering LDL-cholesterol in the blood^8,9^. Due to more stability to oxidation than sunflower oil^10^, the amaranth seeds oil has potential to development of healthy products with a longer shelf life. Amaranth seeds represent an important source of folate and starch^11^. The starch of amaranth consists mainly of amylopectin, 7.8–34.3%^12^, while the amylose content is lower: 5–7%^13^. Some studies have shown that the replacement of wheat flour (WF) with amaranth (AF) not only enhances its chemical composition but also improves the properties of the flour, dough and bread's quality attributes. The replacement of refined wheat flour with amaranth flour significantly enhanced the nutritional bread value resulting in high protein, mineral, fiber, and myo-inositol phosphates content of the final product^14,15^. The composition of amaranth fibers (4.9–13.5%) is similar to that of vegetables and legume seeds^16^. Morphology structure knowledge of amaranth seeds plays a key role in obtaining enriched fractions in certain compounds because the quality of the baked products is correlated with the compounds from flour particle size. Amaranth seeds present different external and internal morphology which lead to differences in nutrient distribution^17^. The amaranth seed perisperm (full of polygonal starch cells) is high and nutrient-rich, being wrapped by a peripheric embryo^9,17^. Amaranth starch contains a big different ratio between amylose (2–12%) and amylopectin content (90–98%), which will affect the physical and chemical properties of starch^18^. The amylose content from amaranth seed is associated with functional properties, such as rheology (pasting and thermal characteristics) and texture properties, amaranth starch presenting good gelatinization properties^19^. Amaranth seeds includes also a high content of non-starch polysaccharides, like dietary fibers^20^. This morphology can affect the nutrient concentration distribution during the milling process due to the breakage of the seeds^17^ and can influence the dough’s rheological behaviour during bread-making stages and the quality attributes of baked products, respectively.

The replacement of gluten in bread with non-gluten raw materials represents a big challenge because it is the dough structure-building protein. Its dilution affects the dough’s property through the kneading, leavening, and baking process. The dynamic rheological methods simulate the viscoelastic behavior of the composite dough which is formed by the interactions between amaranth fractions and gluten matrix from wheat flour^21^. AF replacement produced modifications on the rheological and textural parameters of wheat dough due to the gluten dilution effect^14,15,22–24^. The gas retention in leaven bread is influenced by the fibers and starch content from amaranth fractions. The interactions between particle sizes and amaranth flour percentage which replaced wheat flour led to a decrease in volume, porosity, and elasticity, and an increase in firmness, with the increase of amaranth flour amount^15,25^.

There is a lack of research about the replacement of wheat flour with different amaranth particle sizes at various levels, regarding how the particle size features can influence the physicochemical characteristics of wheat flour, dough dynamic state, bread physical, and sensorial profile. Starting from those presented, we believed that the research on the molecular features and microstructure of amaranth flour fractions, chemical composition of composite flour formulations, dynamic dough rheological properties, and bread characteristics is needed because provides valuable information from nutritional and economic standpoints.

## Materials and methods

### Materials

Wheat flour (WF) with an extraction rate of 65% (2020 harvest) provided a local mill (Mopan, Suceava, Romania), amaranth seeds and salt acquired from the market (SanoVita S.R.L, Vâlcea, România) and fresh *Saccharomyces cerevisiae* yeast (Rompak, Paşcani, România) were used. Amaranth seeds were used for wheat flour substitution, after a grinding according to the protocol reported in our previous studies^24^. Amaranth seeds were grounded with a laboratory grinder (KitchenAid, Whirlpool Corporation, Benton Harbor, MI, USA), sifted for 30 min at 70 HZ amplitude with Retsch Vibratory Sieve Shaker AS 200 basic (Haan, Germany), and separated in three particle size (PS), as: large (L > 300 µm), medium (M > 180 µm, <  300 µm) and small fractions (S <  180 µm).

The WF was analyzed according to the International Association for Cereal Chemistry (ICC)^26^ standard methods (110/1, 105/2, 136, 104/1, 107/1) for the following characteristics: moisture (14.08%), protein (12.45%), fat (1.41%), ash (0.69%), and Falling number index (312 s), while Romanian standard procedure^27^ was used to determine wet gluten (30.00%) and gluten deformation index (6.00 mm). Amaranth seeds proximate composition included: 14.00% moisture, 17.00% protein, 2.00% ash, and 8.00% fat. The proximate composition of the AF fractions was determined and reported in previous work^24^.

### Flours morphological analysis

WF and AF fractions were evaluated regarding their microstructure with VEGA II LSH electron scanning microscopy (Tescan, Brno, Czech Republic), at an acceleration tension of 30 kV. Each sample was coated with a double-sided adhesive carbon band before being scanned and collected at 2000×, 1000×, 500×, and 100× magnifications.

### Assessment of functional groups from flours' FT-IR spectra

The presence of various functional groups was assessed by Thermo Scientific Nicolet iS20 (Massachusetts, USA) spectrometer equipped with an attenuated total reflectance ATR accessory. The FT-IR spectra of the WF and AF fractions were achieved at a spectral resolution of 4 cm^−1^ by 32 scans recorded between 650 and 4000 cm^−1^, the graphs being evaluated with OMNIC software (9.9.549 version, Thermo Fisher Scientific, Waltham, MA, USA) based on previous studies, in order to identify the molecular characteristics of the flours^28^.

### Composite flour formulations

Refined wheat flour was substituted in the proportion of 0%, 5%, 10%, 15%, and 20% with each amaranth fraction obtained through the milling and sieving process (L, M, and S) and mixed with a Yucebas Y21 mixer (Izmir, Turkey), in order to obtain the following composite flour formulations coded as: AL_5, AL_10, AL_15, AL_20, AM_5, AM_10, AM_15, AM_20, AS_5, AS_10, AS_15, and AS_20. The sample with 0% AF was used as a control.

### Physico-chemical characterization of the formulated flours

The formulated flours were analyzed according to the International Association for Cereal Chemistry (ICC)^26^: moisture content (ICC 110/1), protein content, determined with a Kjeldahl device (VELP Scientifica, Usmate Velate (MB), Italy), and calculated with a general factor of 6.25 for wheat flour and 5.53 for wheat-amaranth composite flour (ICC 105/2), fat content, determined with the Soxhlet method (VELP Scientifica, Usmate Velate (MB), Italy) (ICC 136), ash content, determined by incineration at 900 °C (ICC 104/1), and total carbohydrate content was calculated by difference, as a percentage of the total mass. The composite flours color parameters were assessed using a colorimeter CR‐400 (Konica Minolta, Osaka, Japan) and CIELAB scale: L*—lightness/darkness (0 for black and 100 for white), a*—the intensity of green (− a* = more green) or red (+ a* = more red), and b*—the intensity of blue (− b* = more blue) or yellow (+ b* = more yellow), and Chroma (C*) was calculated according to Eq. (1).

1 $${C}^{*}= \sqrt{{a}^{*2}+{b}^{*2}}$$

### Dough and bread processing

For breadmaking was used 300 g flour, 5.40 g salt, 90 g yeast, and water (the quantity required to yield a dough consistency corresponding to the C1 torque value of 1.1 N∙m from the Mixolab device). In the first stage, it was obtained a leaven from an entire quantity of water and yeast, and half quantity of flour, that was left for fermentation, 120 min, at 30 °C and relative humidity (85%), in a fermenting chamber (PL2008, Piron, Cadoneghe, Padova, Italy), according to our previous method^24^. When the fermentation operation is over, the fermented leaven is mixed with the second half of the flour and salt in the Kitchen Aid mixer (Whirlpool Corporation, Benton Harbor, MI, USA) for 10 min. The obtained dough was left to ferment in the same conditions for 60 min. The dough was cut in 400 g/piece, molded, placed in aluminum trays for one hour to produce the final fermentation, and baked for 25 min, at 220 °C (oven Caboto PF8004D, Cadoneghe, Padova, Italy).

### Dynamic dough rheology

A Thermo-HAAKE, MARS 40 (Karlsruhe, Germany) with parallel plate-plates geometry was used to determine the dynamic rheological behavior of dough. Dough samples were preliminarily tested for the linear viscoelastic region (LVR), by applied strain sweep tests with strain from 0.01 to 1%, at a constant oscillation frequency of 1 Hz^29^. The flour and water were mixed until reaching the optimum consistency, in order to obtain the dough, and let it rest 5 min before testing^30^. The elastic modulus (G′), viscous modulus (G″), and loss tangent (tan δ) were determined by applied frequency sweep test from 0.01 to 20 Hz, at a constant strain of 0.10% and the values were considered at 1 Hz. To determine the maximum gelatinization temperature (T_max_), considered at the maximum G' value, the temperature sweep test in which dough samples were heated from 20 to 100 °C at a rate of 4 °C per min was performed.

To evaluate the dough resistance to stress during the bread-making was applied a creep-recovery test with a constant shear stress of 25 Pa, at 20 °C, for two time periods, 60 s as creep time under stress and 180 s as recovery time after stress removed^30,31^. The compliance parameter was determined with Eq. (2), where J (Pa^−1^) represent compliance, γ, the strain, and *σ*, the constant stress (Pa^−1^) applied:

2 $$\mathrm{J }\left(\mathrm{t}\right)= \frac{\upgamma (\mathrm{t})}{\upsigma }$$

The creep-recovery test data were submitted to nonlinear equations of Burgers model, by using Eq. (3) for creep phase and Eq. (4) for recovery phase^32^.

3 $$\mathrm{J }\left(\mathrm{t}\right)= {\mathrm{J}}_{\mathrm{Co}}+ {\mathrm{J}}_{\mathrm{Cm }}\left(1-\mathrm{exp}\left(-\frac{\mathrm{t}}{{\uplambda }_{\mathrm{C}}}\right)\right)+\mathrm{ t}/{\upmu }_{\mathrm{Co}}$$

4 $$\mathrm{J }\left(\mathrm{t}\right)= {\mathrm{J}}_{\mathrm{max}}- {\mathrm{J}}_{\mathrm{Ro }}-{\mathrm{J}}_{\mathrm{Rm}}(1-\mathrm{exp}\left(-\frac{\mathrm{t}}{{\uplambda }_{\mathrm{R}}}\right)$$

where J_io_ (Pa^−1^) = instantenous compliance; J_im_ (Pa^−1^) = retarted elastic compliance or viscoelastic compliance; t (s) = phase time; λ_i_ (s) = retardation time; µ_Co_ (Pa s) = zero shear viscosity; J_max_ (Pa^−1^) = maximum creep recovery. The recovery compliance, J_r_ (Pa^−1^) is determined from the sum of J_Ro_ and J_Rm._

### Bread quality parameters analysis

Bread physical properties were measured in triplicate, two hours after baking, in agreement with the Romanian procedure^27^ in terms of loaf volume, and porosity. Loaf specific volume (cm^3^) was found by employing the seed displacement procedure. Porosity was calculated based on a sample cylinder volume (60 mm height and 45.50 mm diameter).

Color analysis was determined after the bread was cut in half and the crumb and the crust color were measured in triplicate by using a CR-700 colorimeter (Konica Minolta, Tokyo, Japan). The bread color characteristics measured were luminosity (L*), red-green intensity (a*), and yellow-blue intensity (b*), whereas the Chroma (C*) was calculated using Eq. (1).

The bread was cut into slices of 50 mm thickness for the texture properties determination (in triplicate) by using a TVT-6700 texture analyzer (Perten Instruments, Hägersten, Sweden). A 2.5 cm cylindrical stainless-steel probe was used to compress twice the sample to a penetration distance of 20% of its depth, at a test speed of 1.0 mm/s, trigger force of 5 g, with an interval of 15 s between compressions. Firmness, springiness, gumminess, and cohesiveness were registered.

### Bread sensorial analysis

The sensory characteristics of the bread were evaluated using an overall acceptability descriptor based on a 9-point hedonic scale in which the following were evaluated: Overall appearance and shape, Surface and properties of the crust, Structure and elasticity of the crumb, Smell, and Taste. Samples were coded with randomly selected four-digit numbers. A 13 semi-trained panelists in sensory analysis who were experts in the field of bread technology assessed the sensory attributes of the bread trials. The panelist scored for different, 1: extremely dislike, 5: neither like nor dislike, and 9: extremely like, and between each assessment, the water and crackers have been consumed.

### Data statistical analysis

Statistical software SPSS 25.0 (trial version) (IBM, New York, NY, USA) was used to calculate the means values and standard deviations for the quantitative data (https://www.ibm.com/products/spss-statistics). Statistically significant differences between parameters were determined by two-way analysis of variance with Tukey’s test at P ≤ 0.05 significance level. A principal component analysis (PCA) was performed to observe the similarities or dissimilarities between the evaluated parameters and formulated samples’ chemical constituents, dough rheological properties, and bread features.

## Results

### Microstructure of flours

**Figure 1:**
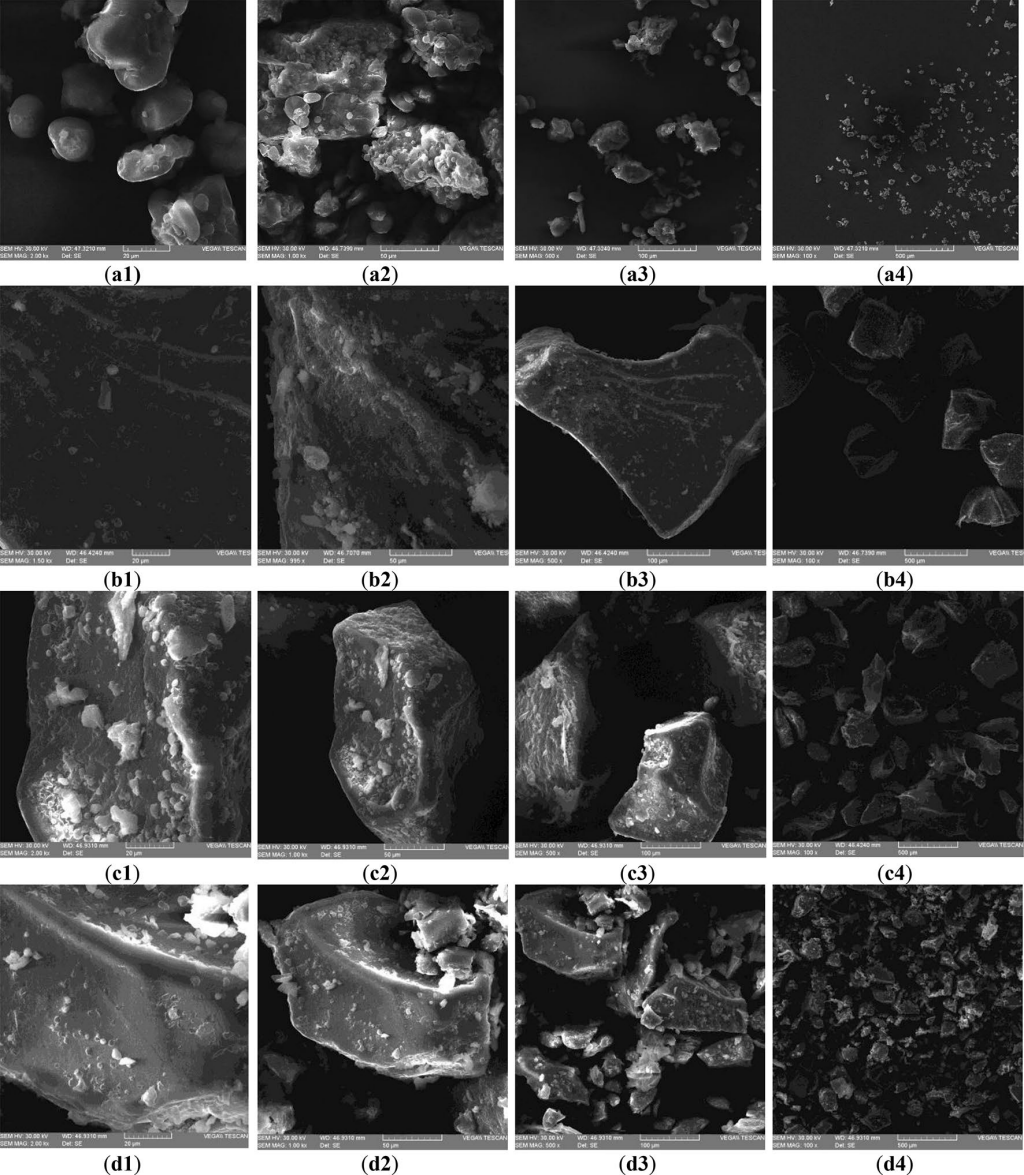
Microstructure of wheat flour (**a1**–**a4**) and amaranth flour large particle size (**b1**–**b4**), medium particle size (**c1**–**c4**), and small particle size (**d1**–**d4**) at different magnifications: ×2000 (1), ×1000 (2), ×500 (3) and ×100 (4).

The scanning electron micrographs of WF and AF fractions are presented in Fig. 1. As can be seen, the amaranth starch granules were round, oval, and irregular in shape.

### Fourier transforms infrared spectrometry analysis of flours

**Figure 2:**
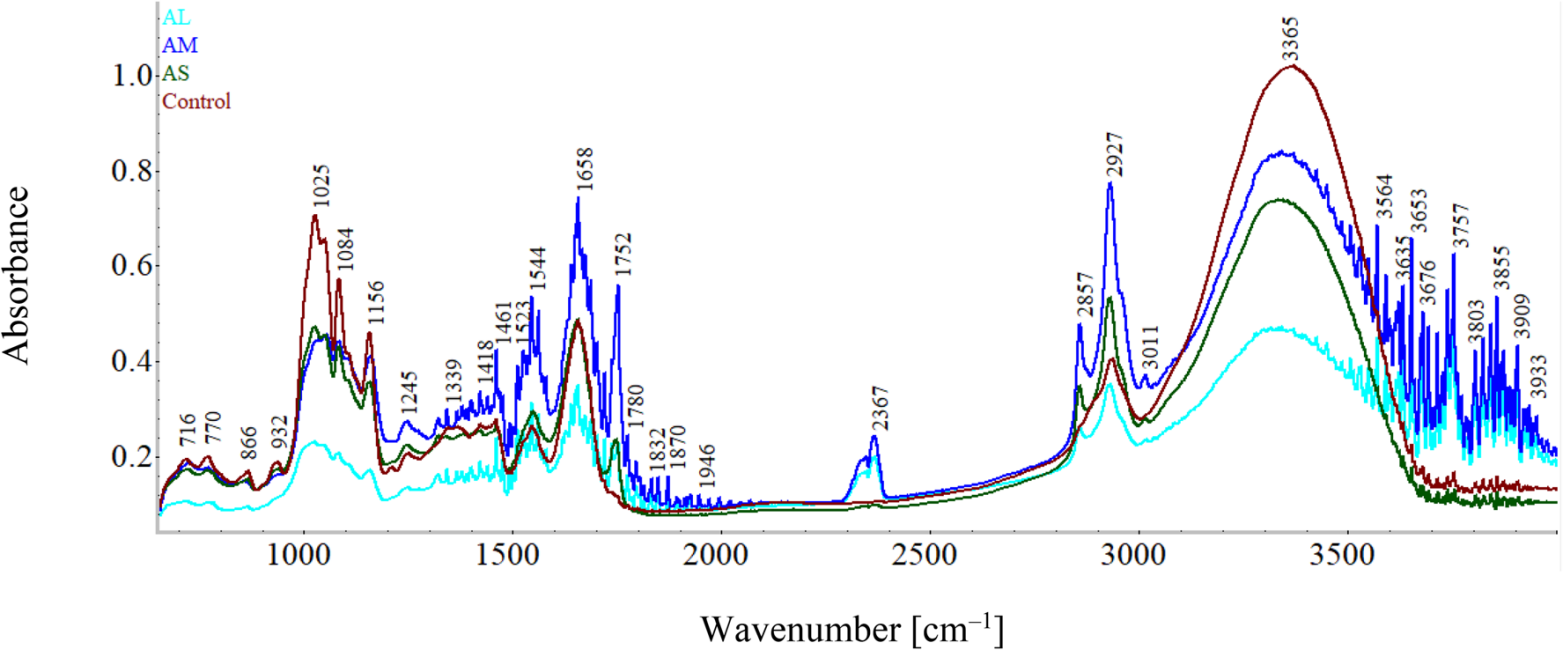
FT-IR spectra of wheat flour and amaranth flour particle sizes, large (AL), medium (AM) and small (AS).

The spectra of the wheat flour and amaranth flour particle size are shown in Fig. 2. The signal heights of samples spectra regarding different types of bonds stretching on the spectrums of wheat flour and amaranth fractions were interpreted according to literature data^33^.

### Physico-chemical properties of composite flours

**Figure 3:**
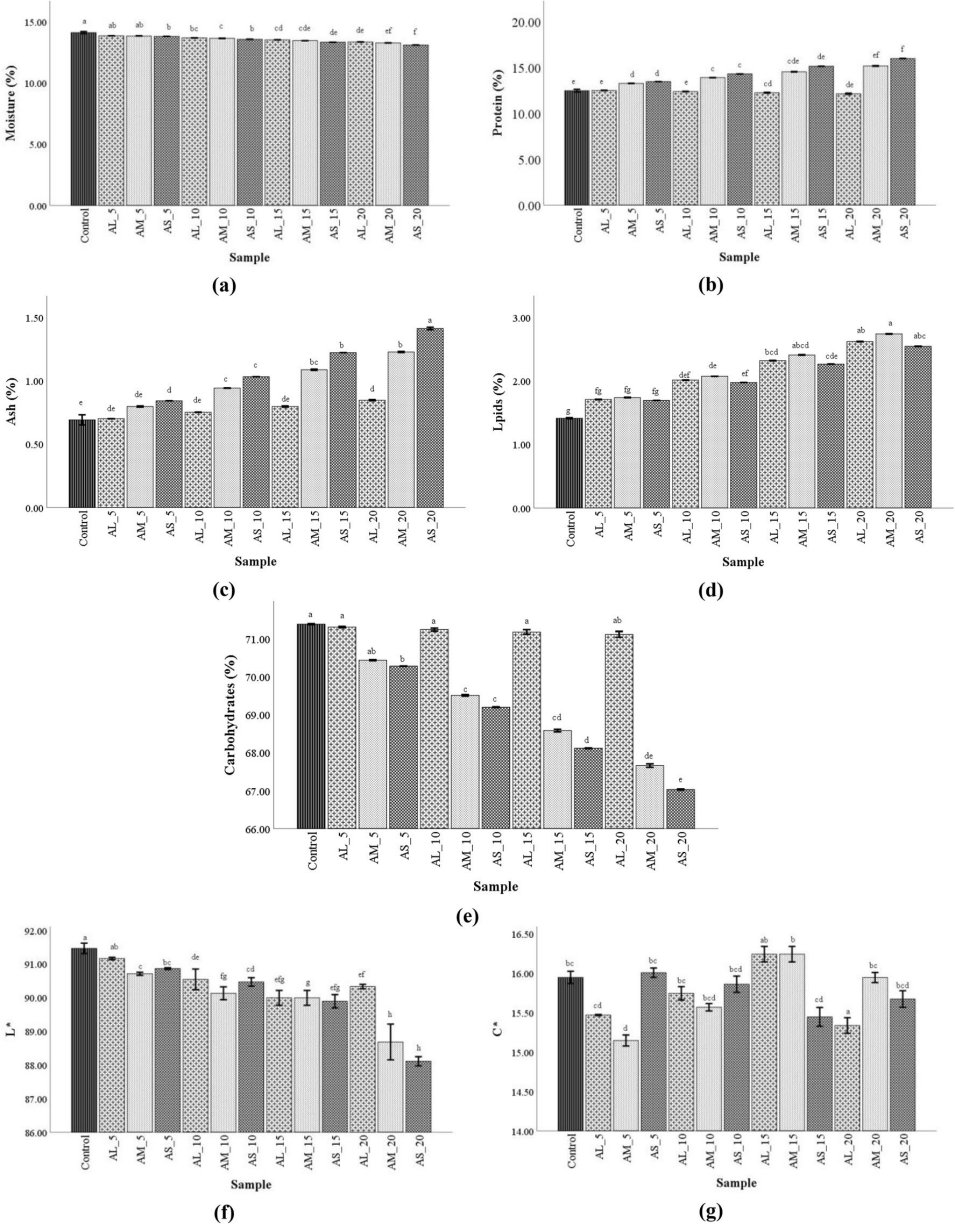
Physico-chemical properties of composite flour formulations with different amaranth flour particle sizes, large (AL), medium (AM), and small (AS) and wheat flour replacement levels (5, 10, 15 and 20%) (**a**–**h**, mean values followed by different letters are significantly different, P <  0.05). L*—Lightness; C*—Chroma.

The physico-chemical properties of composite flour formulations are presented in Fig. 3. The results revealed that the nutrient compositions were markedly influenced by the AF particle size as well as WF replacement level.

As per the graph, moisture (Fig. 3a) was decreased when PS become finest and AF replacement rose, being lower than in the control. The effect of PS and WF replacement was considerable on the protein content of the formulation (Fig. 3b). The finest AF particles size (S and M) led to an enhancement of composite flour protein content by raising the WF replacement, whilst the larger fractions (L) decreased the protein content of these flours. The ash content of wheat-amaranth composite flour (Fig. 3c) increased proportionally with the successive replacement of the WF and with a decrease in particle size. The lipid content of formulated flours (Fig. 3d) was significantly affected when the level of replacement increased, with all samples presenting higher values than the control. Regarding the particle size influence, the lipid content in composite flours increased in the following order: S <  L <  M. Additionally, WF replacement with AF led to a significant decrease in the carbohydrates content of composite flours (Fig. 3e), being successively decreased with the decreased particle size. The color parameters of composite flour were measured on the lightness (L*) and chroma (C*) color scale, and results are given in Fig. 3f,g, being observed significant differences (P <  0.05). A significant increase in the C* parameter with the increase of WF replacement and with a decrease in particle size was observed.

### Dynamic dough rheological properties

Dynamic dough rheological properties were significantly (P < 0.05) influenced by the AF particle size and WF replacement level (Table 1).

**Table 1:** Elastic and viscous moduli, loss tangent, maximum gelatinization temperature, and creep-recovery compliance of bread samples with different amaranth flour particle sizes, large (L), medium (M) and small (S) and wheat flour replacement levels (5, 10, 15 and 20).

Sample	G*'* (Pa)	G*''* (Pa)	tan δ (adim.)	T_max_ (°C)	Jc_max_ (10^−^5 Pa^−1^)	Jr_max_ (10^−5^ Pa^−1^)
Control	26,370.00 ± 70.15^a^	9488.00 ± 60.00^a^	0.3598 ± 0.00^c^	82.74 ± 0.49^a^	24.46 ± 0.04^bc^	16.62 ± 0.00^w^
AL_5	33,400.00 ± 3730.00^by^	11,635.50 ± 302.40^bxy^	0.3517 ± 0.01^byz^	81.94 ± 0.04^cz^	20.45 ± 0.91^abxy^	13.30 ± 0.05^az^
AM_5	33,010.00 ± 2970.00^by^	11,407.00 ± 1113.00^bxy^	0.3465 ± 0.0.00^ayz^	79.77 ± 0.91^bz^	13.54 ± 0.07^axy^	8.63 ± 0.04^cz^
AS_5	23,245.00 ± 1785.00^axy^	8066.00 ± 805.00^axy^	0.3485 ± 0.00^byz^	79.14 ± 0.26^cz^	32.56 ± 2.53^cxy^	20.00 ± 0.05^bz^
AL_10	27,350.00 ± 1250.00^bxy^	9977.00 ± 383.00^bxy^	0.3649 ± 0.00^bzw^	79.51 ± 1.46^cy^	16.63 ± 2.14^abx^	9.67 ± 0.05^ax^
AM_10	30,610.00 ± 830.00^bxy^	10,305.00 ± 235.00^bxy^	0.3367 ± 0.00^azw^	77.96 ± 0.05^by^	16.30 ± 2.57^ax^	13.21 ± 0.05^cx^
AS_10	28,510.00 ± 1480.00^axy^	9943.50 ± 406.00^axy^	0.3490 ± 0.00^bzw^	79.51 ± 0.78^cy^	24.68 ± 6.18^cx^	19.50 ± 0.05^bx^
AL_15	34,345.00 ± 3005.00^by^	11,925.00 ± 1155.00^by^	0.3478 ± 0.00^by^	78.79 ± 0.26^cy^	22.71 ± 4.04^abxy^	6.67 ± 0.27^ay^
AM_15	34,625.00 ± 155.00^by^	11,505.00 ± 45.00^by^	0.3322 ± 0.00^ay^	78.54 ± 0.46^by^	16.95 ± 3.57^axy^	13.44 ± 0.05^cy^
AS_15	24,450.00 ± 1810.00^ay^	8427.00 ± 842.00^ay^	0.3447 ± 0.00^by^	79.34 ± 0.17^cy^	23.93 ± 0.30^cxy^	18.17 ± 0.05^by^
AL_20	58,840.00 ± 2280.00^bz^	19,010.00 ± 850.00^bz^	0.3230 ± 0.00^bx^	78.97 ± 0.21^cy^	23.29 ± 2.40^abxy^	11.24 ± 0.05^ay^
AM_20	45,375.00 ± 825.00^bz^	14,405.00 ± 215.00^by^	0.3175 ± 0.00^ax^	78.43 ± 0.04^by^	18.15 ± 1.40^axy^	13.54 ± 0.05^cy^
AS_20	29,660.00 ± 100.00^az^	9786.00 ± 49.00^az^	0.3299 ± 0.00^bx^	80.61 ± 0.48^cy^	25.50 ± 0.35^cxy^	16.77 ± 0.05^by^
**Two-way ANOVA *p* value**						
FI	P < 0.0001	P < 0.0001	P < 0.0001	P < 0.0001	P = 0.0410	P < 0.0001
FII	P < 0.0001	P < 0.0001	P < 0.0001	P < 0.0001	P < 0.0001	P < 0.0001
F IxFII	P < 0.0001	P < 0.0001	P = 0.0400	P < 0.0001	P < 0.0001	P < 0.0001

FI: amaranth flour particle size; FII: amaranth flour addition level; mean followed by the same alphabets in each column are not significantly different (P > 0.05); the first (a-c) and second (x-w) letter in each column indicates particle size and replacement level, respectively. G′: elastic modulus; G″: viscous modulus; tan δ: loss tangent; T_max_: maximum gelatinization temperature; Jc_max_, Jr_max_: maximum creep-recovery compliance.

The elastic modulus was significantly (P < 0.001) higher when WF replacement increased in comparison with control, being highest when large fractions replaced WF. All dough samples, corresponding to a predominant viscoelastic nature behavior, G′ > G″. Significant differences (P <  0.01) on samples' loss tangent (tan δ) were recorded with the increase of WF replacement with above 10% AF, leading to a gradual decrease of this parameter, while the replacement between 5 and 10% did not have a significant effect on tan δ. Regarding PS, significant differences were registered only between medium particle size and the other two PS (L and S). Particle size influenced Tmax due to WF replacement with AF in comparison with the control, but differences between particle sizes were observed only in the samples where was incorporated medium PS. Replacement level significantly influenced this parameter when was up to 10%. Maximum creep compliance (Jc_max_) presented higher values in samples that were replaced with small PS, followed by large PS, while in samples where was incorporated medium PS, the creep compliance was lowest. The same trend was observed for all the replacement levels, which led to higher dough extensibility. Maximum creep recovery (Jr_max_) was influenced significantly by both factors, PS and replacement level. Usually, this parameter tends to decrease when the WF replacement level with AF increased, and regarding particle size, it decreases in the following order: M < L < S.

### Bread evaluation

#### Physical properties

**Figure 4:**
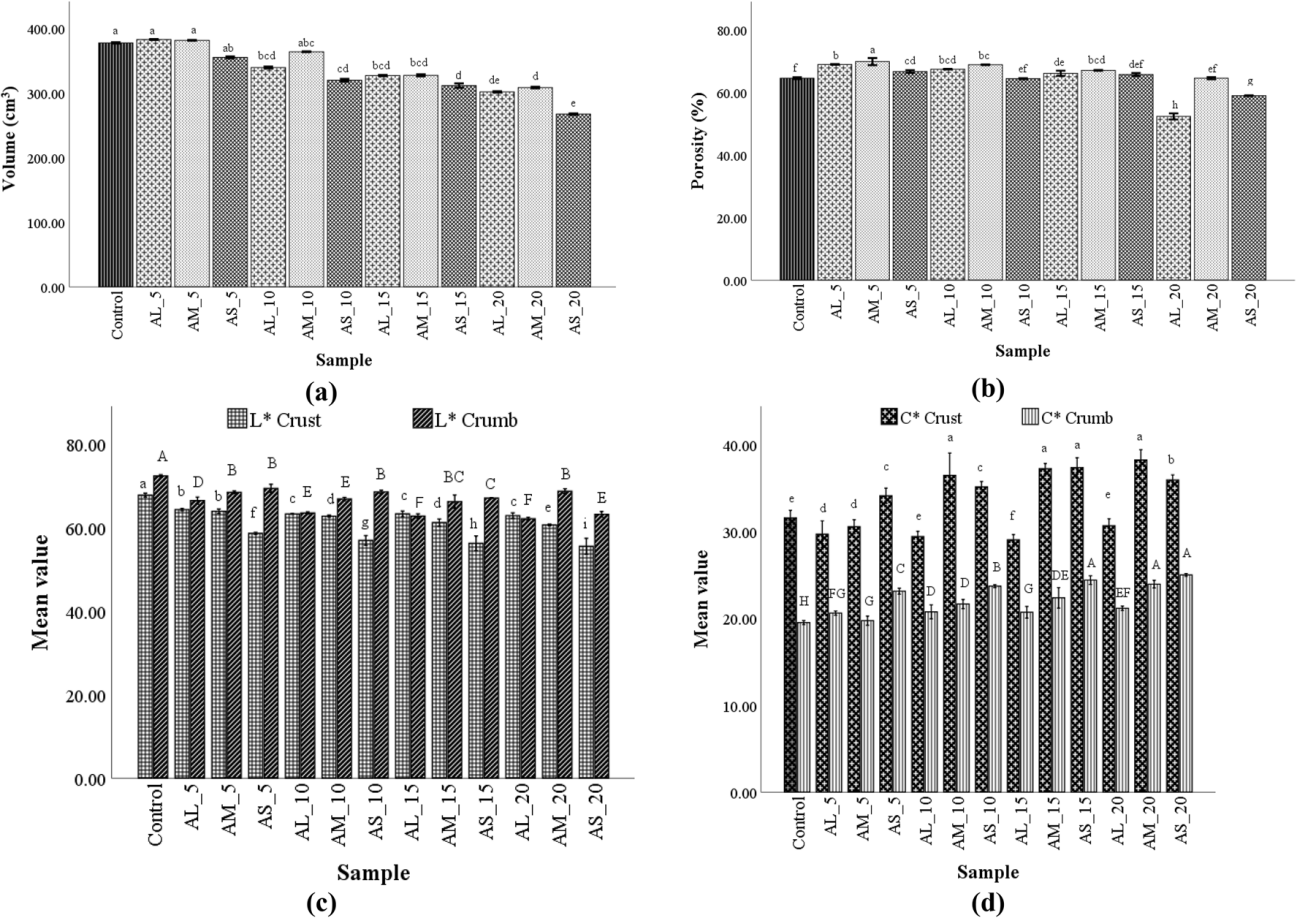
Physical and color parameters of bread samples with different amaranth flour particle sizes large (AL), medium (AM), and small (AS) and wheat flour replacement levels (5, 10, 15 and 20%); Means in the same column with different letters indicate significantly difference (P <  0.05): (**a**–**e**) for bread volume, porosity, and L*, C* Crust; and A–H for L* and C* Crumb. L*—Lightness; C*—Chroma.

Bread physical parameters were significantly influenced by the PS and WF replacement with AF. As Fig. 4a shows, the bread volume was lowest in the sample in which WF was replaced with small PS, and highest in bread with medium PS, followed by large PS. Regarding replacement level, the decrease in bread volume was more accentuated for the samples with higher levels of AF. The porosity (Fig. 4b) of the all bread-based on AF fractions, at replacement between 5 and 15% was higher than wheat flour bread, whilst, the 20% replacement significantly decreased bread porosity. Particle size influences crumb porosity as the following trend: M, L, and S.

#### Bread crumb and crust colour

The lightness (L*) and chroma (C*) for the bread crust-crumb varied depending of AF particle sizes and replacement levels on WF (Fig. 4c,d). Crust lightness (L*) increased gradually with the decrease of PS and decreased with the AF replacement level. Regarding crumb lightness, followed the same trend, depending on formulated factors, PS, and replacement level. Crust chroma presented an increase with the raise of replacement level, while PS lead to an increase of bread crust chroma in the following order: L < M < S. Crumb C* presented an increase compared to control when the replacement level of WF raise, whilst regarding particle size influence, C* tend to increase with the decrease of PS.

#### Textural parameters

Bread texture parameters have a direct influence on consumer perception and choice. The effect of PS and WF replacement levels with AF on bread texture shows that both factors significantly affected all the textural parameters (Table 2). Crumb firmness of bread regarding PS, increased in the following order: M < Control < L < S. Bread firmness also increased gradually with the increase of WF replacement level. Crumb springiness and cohesiveness were not affected by the AF particle size but presented significant differences between samples with 5–10% and samples with 15–20%. Crumb cohesiveness and chewiness significantly increased (P <  0.05) in all breads being higher than control, except for bread with 5 and 10% medium PS which presents lower values than control bread.

**Table 2:** Textural parameters of bread samples with different amaranth flour particle sizes, large (L), medium (M) and small (S) and wheat flour replacement levels (5, 10, 15 and 20).

Sample	Firmness (N)	Springiness (adim.)	Cohesiveness (adim.)	Gumminess (N)	Chewiness (J)
Control	7.71 ± 0.04^a^	1.3458 ± 0.19^c^	0.7664 ± 0.02^c^	602.30 ± 13.92^a^	602.30 ± 13.92^a^
AL_5	8.19 ± 0.11^bx^	1.2475 ± 0.00^ay^	0.8578 ± 0.01^bw^	623.74 ± 8.77^cx^	623.74 ± 8.77^cx^
AM_5	6.06 ± 0.02^ax^	1.1544 ± 0.00^ay^	0.7411 ± 0.00^aw^	458.35 ± 4.44^ax^	458.35 ± 4.44^ax^
AS_5	9.19 ± 0.73^cx^	1.2073 ± 0.00^ay^	0.8850 ± 0.00^cw^	678.22 ± 5.85^bx^	678.22 ± 5.85^bx^
AL_10	12.10 ± 0.02^by^	1.1319 ± 0.01^ay^	0.7305 ± 0.00^bz^	894.00 ± 7.14^cy^	894.00 ± 7.14^cy^
AM_10	6.19 ± 0.02^ay^	1.0527 ± 0.05^ay^	0.7197 ± 0.00^az^	454.64 ± 3.81^ay^	454.64 ± 3.81^ay^
AS_10	12.10 ± 0.02^cy^	1.1453 ± 0.03^ay^	0.8650 ± 0.00^cz^	743.70 ± 43.98^by^	743.70 ± 43.98^by^
AL_15	21.12 ± 0.29^bz^	1.0000 ± 0.00^az^	0.6930 ± 0.00^by^	1445.94 ± 5.56^cz^	1445.94 ± 5.56^cz^
AM_15	12.64 ± 0.69^az^	1.0000 ± 0.00^az^	0.6930 ± 0.00^ay^	764.03 ± 4.84^az^	764.03 ± 4.84^az^
AS_15	21.57 ± 0.40^cz^	1.0015 ± 0.00^az^	0.6930 ± 0.01^cy^	1070.65 ± 0.65^bz^	1070.65 ± 0.65^bz^
AL_20	28.43 ± 0.67^bw^	0.9985 ± 0.00^ay^	0.6732 ± 0.00^bx^	1950.93 ± 18.68^cw^	1950.93 ± 18.68^cw^
AM_20	28.43 ± 0.67^aw^	0.9980 ± 0.00^az^	0.6732 ± 0.00^ax^	938.21 ± 2.77^aw^	938.21 ± 2.77^aw^
AS_20	32.89 ± 0.02^cw^	0.9988 ± 0.00^az^	0.6400 ± 0.02^cz^	1112.50 ± 7.50^bw^	1112.50 ± 7.50^bw^
**Two-way ANOVA *p* value**					
FI	P < 0.0001	P < 0.0001	P < 0.0001	P < 0.0001	P < 0.0001
FII	P < 0.0001	P = 0.2600	P < 0.0001	P < 0.0001	P < 0.0001
FIxF II	P < 0.0001	P = 0.4460	P < 0.0001	P < 0.0001	P < 0.0001

FI: amaranth flour particle size; FII: amaranth flour replacement level; mean followed by the same alphabets in each column are not significantly different (*p* > 0.05), the first (a-c) and second (x-w) letter in each column indicates particle size and replacement level, respectively.

#### Sensory evaluation

**Figure 5:**
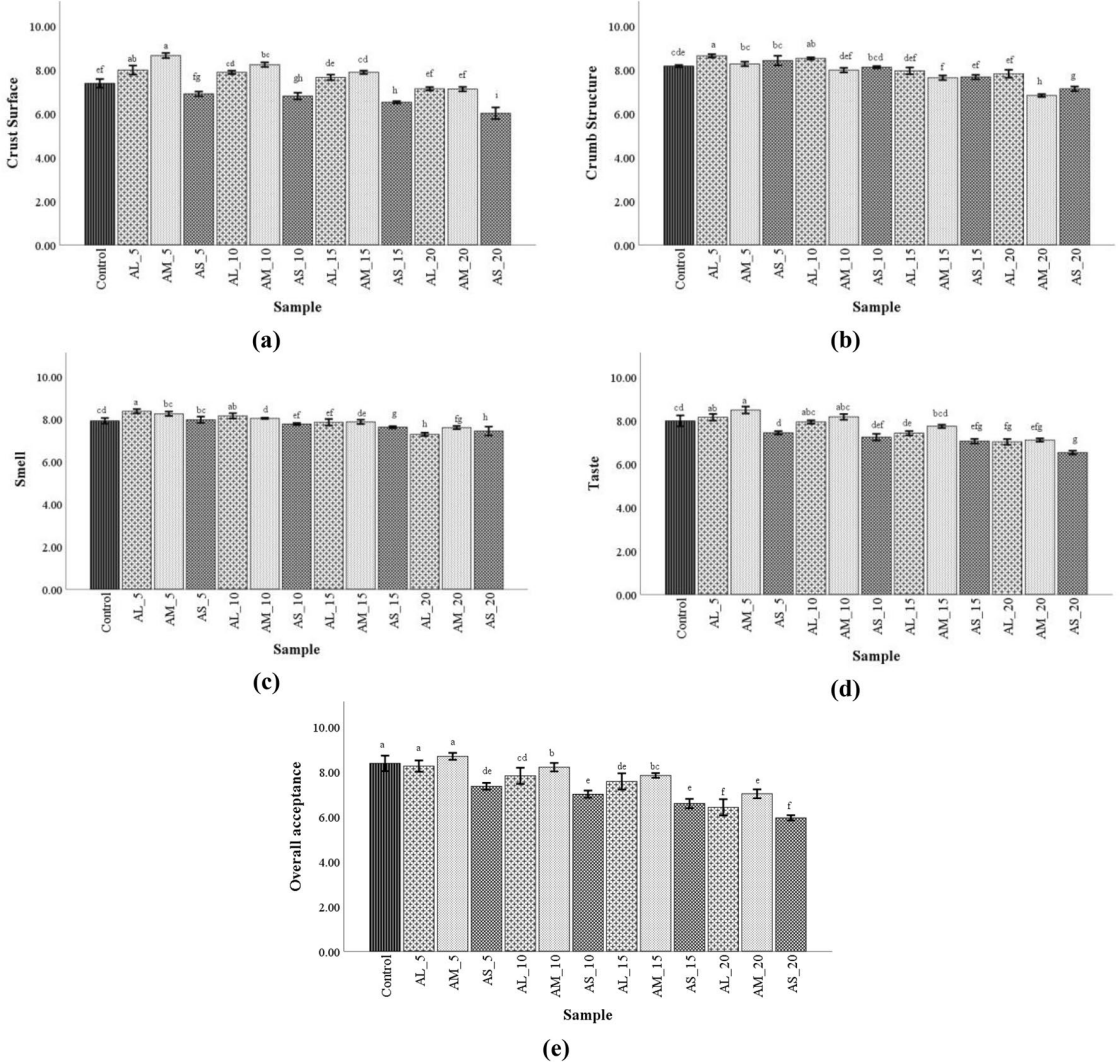
Sensory characteristics score of bread samples with different amaranth flour particle sizes, large (AL), medium (AM), and small (AS) and wheat flour replacement levels (5, 10, 15 and 20%) (**a**–**h**, mean values followed by different letters are significantly different, P <  0.05).

Sensory evaluation results revealed some improvements regarding crust surface, crumb structure, smell, taste, and overall acceptance for bread which contains medium and large particle sizes up to 10%, compared to control (Fig. 5a–e). For bread with a small AF particle size, for all replacement levels were observed a decrease in sensorial acceptance in comparison with bread control.

#### Relations between assessed characteristics

By applying Pearson's correlation analysis between assessed characteristics, a series of siginificant (P < 0.05) correlation coefficients (0.56 ˃ r < 0.98) was found. Flours humidity was strongly positive correlated with loss tangent (r = 0.67), bread springiness (r = 0.78), gumminess (r = 0.61), bread volume (r = 0.95) and bread overall aceptability (r = 0.65), bread taste (r = 0.78) and crumb structure (r = 0.81). Instead this physical parameter of flour was nevatively associated with bread gumminess and chewiness (r = ‒ 0.58). In this way, it seems that flour humidity is a good indicator for flour quality which has direct correlation with dough and bread properties. High positive correlation were found between flour lipids and bread firmness (r = 0.66) and gumminess (r = 0.67), and elastic modulus (G*'*) (r = 0.58), while lipids are negatively associated with loss tangent (r = ‒ 0.70), bread volume (r = ‒ 0.85), bread springiness (r = ‒ 0.82), bread cohesiveniss (r = ‒ 0.72), and with all sensorial characteristics: structure (r = ‒ 0.80), smell (r = ‒ 0.73), overall aceptability (r = ‒ 0.53). Regarding the bread texture, it was found significant (P < 0.05) correlation with dough rheology and consumer accpetance of final product. Bread firmness is positive correlated with elastic modulus (r = 0.67) and viscous modulus (r = 0.69), while with bread volume (r = ‒ 0.58), bread porosity (r = ‒ 0.70), and bread oberall aceptability (r = ‒ 0.56) is negatively associated. Bread springiness is positive correlated with loss tangent (r = 0.56), bread volume (r = 0.67), and bread structure (r = 0.74). Bread gumminess and chewiness are positve associated with elastic modulus (r = 0.59), viscous modulus (r = 0.60), while in a negative way is associated with bread volume (r = ‒ 0.65), bread porosity (r = ‒ 0.74), and sensory characteristics of bread: overall aceptability (r = ‒ 0.64).

**Figure 6:**
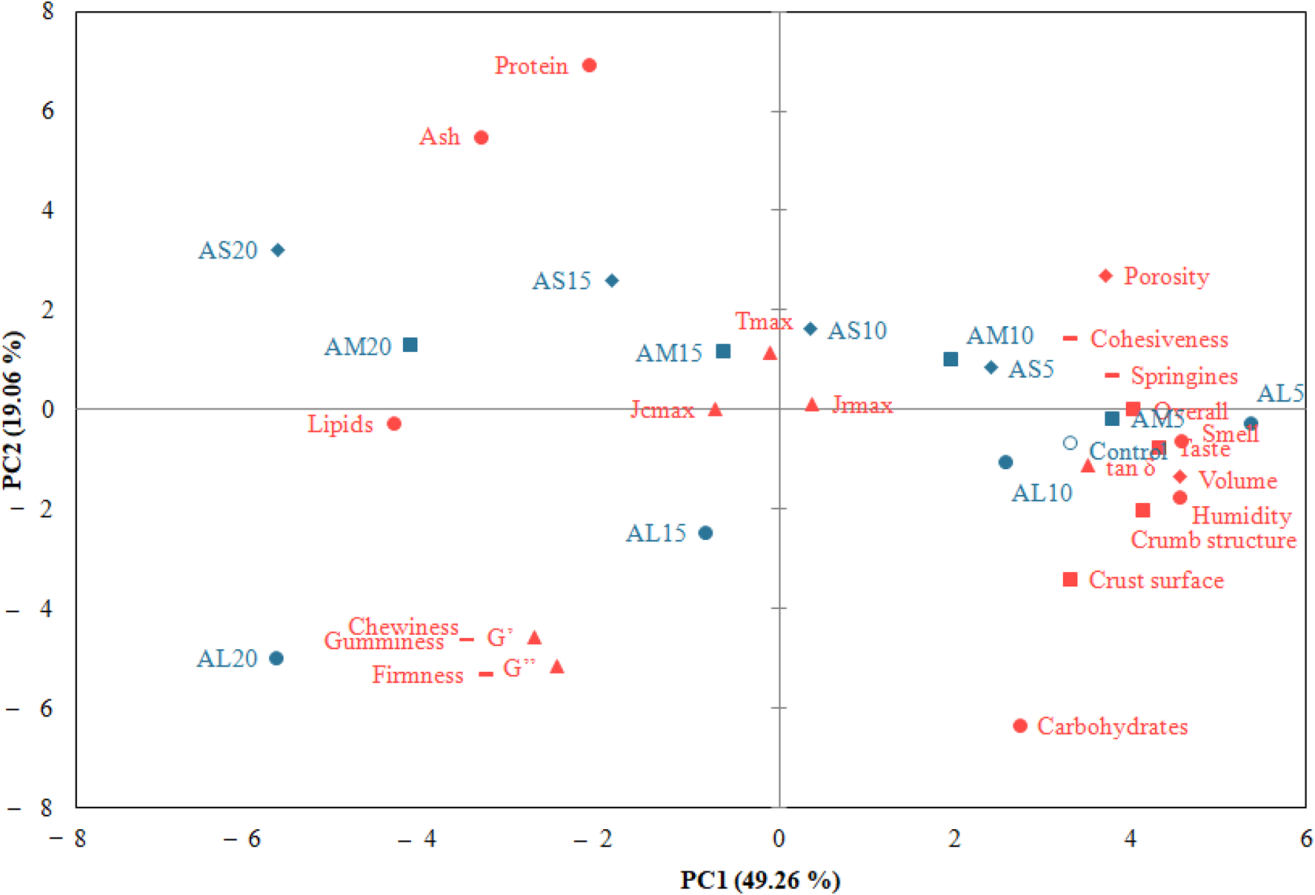
Principal component analysis bi-plot revealing the relationships between the proximate composition, dough dynamic rheological parameters, bread physical, textural and sensory characteristics, and formulated samples with different amaranth flour particle sizes, large (AL), medium (AM), and small (AS) and wheat flour replacement levels (5, 10, 15 and 20%).

The principal component analysis (PCA) was used to highlight the similarities or dissimilarities between the determined characteristics (Fig. 6). The loadings of the studied characteristics on the first principal component, PC1 (49.26%), and the second principal component, PC2 (19.06%) described 68.32% of the total variance.

The dough maximum gelatinization temperature (T_max_) and creep-recovery compliance (Jc_max_ − Jr_max_) have a small contribution to the data variation, as is suggested by their position on the graphic, close to the center. Instead closeness of single parameters for example flour humidity, loss tangent (tan δ), bread volume, and sensory characteristics confirms a tight pair correlation, as well as the association between elastic and viscous moduli (G*'*, G*''*), bread firmness, gumminess, and chewiness. The PC1 was associated with flour humidity and lipids, tan δ, bread volume and porosity, bread textural parameters, and bread sensory characteristics while PC2 was associated with flour protein, ash, and carbohydrates, elastic, and viscous moduli (G*'*, G*''*). It can be remarked a high opposition between protein-ash and carbohydrates, bread firmness and porosity, viscoelastic moduli and bread springiness. A strong correlation was observed between wheat flour bread and bread with medium and large PS when WF was replaced at a 5% level (AL_5 and AM_5) and 10%, respectively (AL_10). Composite flour with a 20% AF of medium and small fractions was associated with lipids, ash, and protein, whilst samples with large fractions (AL_20) were associated with viscoelastic moduli, bread firmness, gumminess, and chewiness.

## Discussion

The microstructure helped to understand and visualize structural changes and textural differences on fractions and to determine the appearance and texture of flour fractions and the stability of the final product. Scanning electron (SE) micrographs (Fig. 1) show the microstructure of the wheat flour (WF), and amaranth flour particle size: large (L), medium (M), and small (S). Amaranth starch presents polygonal, angular, or irregular granules that are similar to those of wheat, rice, and maize starches^18^. In the WF, it can be observed a homogeneous mix of starch grains and gluten protein (Fig. 1a1–a4) generated by the milling process caused a consistent release of the protein chain from the starch grains. Due to the fractionation and sieving process, large fractions (Fig. 1a1–a4) are characterized by spherical starch granules, together with a few macro-complexes of starch embedded in the protein matrix. In medium particle sizes (Fig. 1b1–b4) starch has rounded shapes, while in small fractions (Fig. 1d1–d4), amaranth is more compact and is presented as a mix of starch grains and protein. In amaranth, the embryo, with cotyledons and radicle, surrounds the perisperm^34^ with starch polygonal cells, which have thin membranes^35^. Amaranth starch from medium and small particle sizes presents higher crystallinity in comparison with wheat starch, which can form amylose lipid complexes^18^. These changes that occur in the morphology of starch, lipids, and protein of amaranth different fractions, can influence composite flour, dough, and bread properties when it will replace wheat flour.

Fourier transform-infrared (FT-IR) is used to characterize materials composition and to observe structural changes influenced by food processing techniques. The band's heights at 716, 770, and 866 cm^−1^ can characterize the substitutions in aromatic rings (aromatic C–H bonds)^36^. The reflectance signal heights of each spectrum at 932–1156 cm^−1^ were recorded from each flour. The intensity ratios of these bands were used as convenient indexes of short-range starch structure^37,38^. The information-rich fingerprint region from 900 to 1500 cm^−1^ contains signals from amylose–lipid complexes present in the whole grain, amide III (1330–1230 cm^−1^), or structural carbohydrates such as starch and cellulose (unsaturated bonds C=C connected to the oxygen atoms O–C=C or the nitrogen atoms N–C=C)^36^. The bands present at 1156, 1084, and 1025 cm^−1^ could give information about the axial deformation vibrations of C–O in alcohols. The band heights were also studied for proteins: amide I (1658 cm^−1^) and amide II (1544 cm^−1^), vibrations, which are the most common vibration to study proteins^39^. Lipids influence can be observed in the bands at 2927, 2857, and 1752 cm^−1^ attributed to C–H stretching vibrational modes of alkylic CH_2_ and CH_3_ groups. The band at 1752 cm^−1^ is typical for the ester carbonyl stretching common of the esterification of fatty acids in the glycerol backbone^33^. Water (OH-stretching vibration) can be observed at bands 3365 cm^−1^ for wheat flour and at 1658 cm^−1^ for amaranth flour medium particle size, this variation among samples is likely due to the milling and sieving process of the samples, being correlated with their humidity. Similar spectra were obtained by Roa et al.^33^.

Wheat-amaranth composite flours presented lower humidity values due to the temperature increase during the process of size reduction, which lead to a drying phenomenon. The observed protein trend of the composite flours varied between 12.11 and 15.95%, which can be explained by the localization of the protein in the embryo (65%) and endosperm (25%) of the seed^2,6,40^. The milling enhances the disentanglement of protein bodies in the cotyledon^41^. Our results are within the range of the literature values^42^. The highest amounts of ash from composite flour are found when the replacement level of AF is maximum and increased gradually with a decrease of PS, indicating that these small fractions could be a deposit for a high amount of minerals. The presence of the high amount of lipids in composite flour is given by the amaranth seeds fat-rich (2 times higher than in other cereals)^43^. Also, a high amount of lipids are observed at flour samples which were replaced with small AF particle size, which could be explained by the localization of this fraction in the embryonic part^43^. The lipids contents from flours lead to changes in bread hardness and aroma^10^. Total carbohydrates content from studied flours decreased when AF fractions became finest and replacement level increased. The desired color of wheat flours for industrial applications is a high value for luminosity and a low value for chroma^44^. The darkening color presented in composite flour with medium particle size can be justified by the fiber and phenols present in the embryo^45^.

Since amaranth flour is gluten-free, its incorporation in wheat flour represents a challenge for dough rheological behavior and final product quality. Dynamic rheology applies small deformation and was used to avoid dough matrix disruption. Both elastic and viscous moduli increased when AF replacement rose, with medium and large PS leading to the highest values, while small PS presented the lowest values. Other reports demonstrated that the increase of elastic modulus is associated with the hydration and swelling process of the amorphous regions of starch granules, which could lead to the future increment of viscous modulus^46^. Furthermore, the complex bonds that are formed between starch granules and amaranth fibers, can contribute to the increase of viscoelastic moduli. Similar results of viscoelastic moduli were found for whole amaranth flour in some studies^46^. Also, the damaged starch that results in the milling process, especially from small fractions, can be correlated with high values of G*'*, leading to firmer doughs and crumb bread (Table 2). Wheat flour replacement with AF had an opposite effect on the T_max_ of composite flour, which has a decreased tendency when raising the replacement level. This phenomenon presumably would be due to the insoluble amylose–lipid complexes that occur during heating starch slurries, which reduce and delay the swelling of starch granules^2^. Creep-recovery compliance is affected by the sugars, protein, or starch from amaranth flours. The hydroxyl groups will interact with a proteic chain that will lead to non-covalent or covalent bonding.

Bread volume reflects the degree of texture weakness. The volume of bread depends on several factors, such as dough viscosity, amylose/amylopectin ratio, and the presence of protein aggregation with an increase in temperature during heating^47^. When the WF replacement level increases, it can be observed a tendency to decrease bread volume, which can be explainable by the lack of gluten protein from amaranth grain^48^. A similar trend was observed by Sanz-Penella et al.^14^ and by Almeida et al.^49^ in bread with wheat flour replaced with amaranth flour. Also, the low amylose content from amaranth flours performs poorly in bread volume. The amylases from starch hydrolyze the amylose which is transformed into maltose, which can be used by the yeast for the production of carbon dioxide, which produces a rise in the dough volume^50^. In samples with medium particle size, a higher volume can be observed in comparison with small particle size. This effect can be due to the high content of protein from these flours, such as albumin, which has the capacity to interact with wheat glutenin protein through disulfide bonds, which does not weaken the gluten network very much^6^. Bread supplement with 5% medium and large PS, presented the highest volume than the wheat bread. Similarly, Mlakar et al.^2^ obtained the highest loaf volume when supplemented wheat and refined spelt flours up to 10% amaranth flour. Bread porosity presented better values for all samples in comparison with control, except for bread with amaranth flour small PS in a concentration of 20%. This improvement could be due to better rheofermentation properties of these composite flours^25^. Similar observations were found by Burisova et al.^51^.

Color is an important parameter that can influence people's acceptance of bread. An increase of WF replacement and decrease of PS imparted darker crumb color and higher yellowness to the bread. These reactions could have been promoted by the sugars and amino acid composition of AF^45^. Sanz Pennella et al.^14^ reported a lower lightness and red nuance of bread when amaranth flour was incorporated. It is generally acceptable to have a darker crust than the crumb, therefore, the chroma of the bread samples was acceptable. The chroma observed could be given by the caramelization or the Maillard reaction during the baking of bread^32^. Also, lysine and other amino acids from amaranth flour react with the reducing sugars, favored by the high temperature, leading to bread darkness^52^.

Firmness is the essential mechanical property for solid foods and represents the force necessary to achieve a given deformation^53^. The firmness shows an increased tendency when AF gradually increased in WF, probably due to the dilution effect and the incorporation of fiber through amaranth flour suggesting that the crumb has a firmer and more compact structure. But, for bread supplemented with 5–10% AF medium fractions, the bread firmness was lower than of the control. This effect was also observed in bread supplemented with quinoa fractions^28^. Amaranth flour is rich in dietary fiber and contains albumin that can interact with wheat glutenin through disulfide bonds, which can act as a surface active agent and will maintain the gluten matrix^6,35^. Also, the polar lipids can act as a gas stabilizing agent during breadmaking, leading to an improvement in bread springiness^53^. Some authors found a direct correlation between dough springiness/crumb chewiness and bread firmness^54^.

In the case of chewiness and gumminess, the values decreased with WF replacement level rise with AF, our results being in accordance with the data reported by other authors that incorporated amaranth flour in wheat flour^14,15,35^. Sensory assessment, decisive especially for the development of novel products, highlighted that the loaf has a regular shape, a slight surface roughness, and an acceptable crust color. Compared to control, bread with 5 and 10% of large and medium AF fractions has better scores. For replacements of WF with AF at a higher level than 15% of large and medium PS, the score decreased in comparison with wheat flour. Lorenz et al.^55^ obtained for amaranth addition up to 15% of the following sensory characteristics: nutty, pleasant tasting, texture slightly firm, and a better flavor than of the control bread, but darker crumb. Tosi et al.^56^ used hyper proteic wholegrain and hyperproteic defatted amaranth flour and for addition up to 8% found a better acceptance score. In the findings of other authors^51,57^ the sensory attributes slowly decreased with increasing of amaranth amount, being more pronounced when replacements levels were higher than 15%.

In this research, it has been demonstrated that using yeast for obtaining amaranth flours sourdough can improve bread's physical, textural, and sensorial properties. The difference in crust and crumb properties, taste–smell, and overall acceptance scores according to amaranth flour PS may be due to the natural characteristic sensory properties of each AF particle size or may be due to different factors affecting microbial growth in the fermented dough. The presence of nitrogen sources, maltose, lipids, as well as enzymatic activity, and growth factors (vitamins and minerals) in the substrate can affect the microbial growth in sourdoughs^58^. It was demonstrated by some authors that yeast fermentation was more successful than spontaneous fermentation in improving the sensory properties of amaranth flour bread^59^.

## Conclusions

The use of amaranth flour to replace refined wheat flour in bread formulations is very interesting due to its valuable nutritional profile and thus will meet consumer demands in terms of nutrition. The changes that occur in the morphology of starch, lipids, and protein of amaranth flour fractions exert influence on composite flour, dough rheology, and bread characteristics. The large and medium amaranth flour fractions at the concentration of up to 10% can be used as a partial replacement for wheat flour in bread formulations without negatively affecting the dough behaviour and product sensory properties. At a higher replacement level, precessing difficulties can occur due to high viscosity. Moreover, dough samples with the small particle size show increased resistance to deformation compared to those with medium and large particle sizes. The maximum gelatinization temperature decreased for all fractions and replacement levels. The replacement of wheat flour with different amaranth flour fractions induced various influences on bread characteristics. Sensory assessment, decisive especially for the development of novel products, revealed that refined wheat flour replacement levels up to 10% with large and medium particle fractions of amaranth flour is considered suitable. A significant correlation (P < 0.05) between composite flour nutrients, dough dynamic rheological properties, and bread physical, textural, and sensory characteristics was found. The results of this study represent essential support for future bread-making optimization trials enriched with amaranth flour.
